# Providing supplementary, artificial milk for large litters during lactation: effects on performance and health of sows and piglets: a case study

**DOI:** 10.1186/s40813-015-0008-8

**Published:** 2015-10-09

**Authors:** J. Pustal, I. Traulsen, R. Preißler, K. Müller, T. große Beilage, U. Börries, N. Kemper

**Affiliations:** 1 Institute of Agricultural and Nutritional Sciences, Martin-Luther-University Halle-Wittenberg, Theodor-Lieser-Straße 11, D-06120 Halle (Saale), Germany; 2 Institute of Animal Breeding and Husbandry, Christian-Albrechts-University Kiel, Hermann-Rodewald-Straße 6, D-24118 Kiel, Germany; 3 Education and Research Centre Futterkamp of the Chamber of Agriculture Schleswig-Holstein, D-24327 Blekendorf, Germany; 4 Auf der Beilage 2, D-49632 Essen (Oldb.), Germany; 5 Börries GmbH&Co.KG, Mühlenberg 17, D-49699 Lindern, Germany; 6 Institute for Animal Hygiene, Animal Welfare and Farm Animal Behaviour, University of Veterinary Medicine Hannover, Foundation, Bischofsholer Damm 15 (Building 116), D-30173 Hannover, Germany

**Keywords:** Foster, Litter size, Piglet, Sow, Suckling

## Abstract

**Background:**

One possible way to support raising large litter sizes in pigs is to offer supplementary, artificial milk *ad libitum* in the farrowing pen in addition to the sow’s milk. In order to evaluate the potential use of this method and its effects on performance and health, supplemented (*n* = 60) and control sows (*n* = 60) with their litters were tested over 15 batches in one herd during one year. In the supplemented group (SG), piglets had access to supplementary milk in addition to sow’s milk from their 2^nd^ day of life until weaning (day 27). The litters of SG sows were adjusted to contain as many piglets as the sow had functional teats, whereas in the control group (CG), piglets were set to the number of functional teats minus one, due to animal welfare reasons.

**Case presentation:**

With supplementary milk provision, the weaning of large litters was achieved without any negative impacts on performance and health. On average, 13.5 and 12.4 piglets were weaned in SG and CG, respectively (*P* < 0.05). While average weaning weights (SG: 7.8 kg *v*. CG: 7.8 kg; *P* > 0.05) and average daily weight gain of the piglets (SG: 0.25 kg *v*. CG: 0.25 kg; *P* > 0.05) did not differ, total litter weight was consequently higher in SG than in CG (104.9 kg *v*. 96.7 kg; *P* < 0.001). The average milk replacer intakes were 1.1 kg milk powder per day and batch, and varied significantly between the “warm” and “cold” seasons (1.5 *v*. 0.9 kg milk powder per batch and day; *P* < 0.001). No significant differences in the mortality rate or the occurrence of diarrhoea were detected in the piglets of both SG and CG (*P* > 0.05). With regard to documented medical treatments, facial lesions were treated less frequently in piglets of SG (46 *v*. 32 treatments; *P* < 0.05). There was no effect of supplementary milk on the loss of body weight, backfat thickness and body condition score of the sows (*P* > 0.05).

**Conclusions:**

To summarise, in the presented case offering *ad libitum* supplementary, artificial milk supported the sow in raising large litters by compensating possible negative impacts of high piglet numbers on the weight gain of piglets and the body condition of the sows.

## Background

Increasing litter size has long been a goal in pig production, resulting in highly-prolific sow lines with a large number of piglets born alive [1]. The health and welfare consequences of high litter sizes are of increasing concern, and the ideal management of these large litters poses a challenge for farmers. Although milk production increases with the demand of the piglets [2], sow milk yield usually reaches an individual maximum [3]. This individual maximum has not increased proportionally to the number of piglets born alive over the recent decades, and therefore, the actual amount of milk per piglet has decreased [3]. Reduced colostrum and milk intake leads to negative effects in piglets such as rising pre-weaning mortality [4], and a decreasing average pre-weaning daily weight gain [5]. Moreover, by larger litter size, body weight losses in sows increase [6, 7]. To support the raising of large litter sizes, farmers use different fostering practices, with cross-fostering being the most common method. However, in prolific herds, the total number of available teats on newly farrowed sows may be less than the number of piglets born. Using nursing sows, artificial rearing systems or provision of supplementary milk are the management options currently available to raise these large litters. As the use of nurse sows involves disruption of batch farrowing and the possibility of vertical disease transmission, this practice may not always be favourable. The provision of appropriately formulated liquid milk supplements via automatic milk dispensers may increase nutrient intake and weight gain [8]. In this article, we report on a practical on-farm test on litters with and without supplemented milk via cups directly in the farrowing pens, and possible impacts on sows’ and piglets’ health and performance. In the presented case, litter sizes had to be adjusted for reasons of animal welfare: it was expected, that without milk supplementation, sows are not able to raise large numbers of piglets without negative effects, contrary to animal welfare, for both sows and piglets. Therefore, sows in the supplemented group retained as many piglets as they had functional teats, whereas control sows retained one piglet less than they had functional teats. It was supposed, that under the provision of supplementary milk, sows can manage one piglet more without negative effects for sows and piglets over the whole suckling period.

## Case presentation

### Animals and techniques

**Fig. 1:**
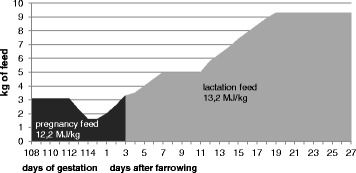
Feeding curve of sows in the supplemented (SG) and control group (CG) in late pregnancy and during lactation. Feed amount (in kg) in relation to day of gestation and day of lactation and the respective feed with specific energy content (in Megajoule per kg feed)

Animals of this report were kept in one herd at the ‘Futterkamp’ Research Centre of the Chamber of Agriculture Schleswig-Holstein in Germany over 15 batches between July 2011 and April 2012. All animals were cared for by the Research Centre staff in accordance with the Federation of Animal Science Societies’ Animal Care Guidelines [9]. Per batch, four sows and their litters were selected for each supplemented group (SG) and control group (CG). In SG, additional artificial milk (Supp-Le-Milk®, Ltd. Boerries, Lindern, Germany) was provided for the piglets in special cups *ad libitum* (Supp-Le-Milk® system, Ltd. Boerries, Lindern, Germany), starting on the 2^nd^ day *post partum*. In total, 60 sows and their progeny (*n* = 1,107 piglets) in SG, and 60 sows and their progeny (*n* = 963 piglets) in CG were analysed in detail. The sows were managed with a 28-day lactation period. Highly prolific sows (Porkuss®, Ltd. ZNVG, Neumünster, Germany) were used, and the litters were products of cross-breeding with Piétrain boars. On average, sows were in their fourth parity (parity class A (1/2): *n* = 28; parity class B (3/4): *n* = 47; parity class C (5–9): *n* = 45). Each sow was randomly assigned to CG or SG, and to one of the eight farrowing pens in the farrowing room, taking their parity numbers into account. The 17 and 23 sows in SG and CG which had had two consecutive lactations within the trial were analysed repeatedly in the same group, respectively. All farrowing pens (5.2 m^2^) were identical and contained an adjustable farrowing crate (115 cm × 62 cm × 168 cm) with heating plates (50 cm × 120 cm), heating lamps (days 1–7 *post partum*) for the piglets outside the crate, and playing materials (ball metal chain for piglets, plastic tube for sows). The farrowing room had its own air-conditioning with dripping ceilings. At higher temperatures, a humidification system was used. The milk provision system was installed with one milk tank in the central hallway and connections to the single milk cups in each farrowing pen by milk lines (four farrowing pens per milk tank). In CG pens, milk cups were locked with dummy plugs. The liquid milk replacer was prepared daily by mixing 120 g of milk powder (Supp-Le-Milk®, Ltd. Boerries, Lindern, Germany) with one litre of warm water (50 to 55 °C), which was then filled into the milk tank. Starting on day two *post partum*, the piglets had *ad libitum* access by pressing a nipple in the cup with their snout. The consumption of supplemented milk was measured daily in total for the four farrowing pens connected to the tank. The milk pipe system was cleaned daily by flushing fresh water through the system and disinfecting it with peracetic acid (Lerasept® Forte, Ltd. Stockmeier Chemie, Bielefeld, Germany). After each weaning, it was cleaned with alkaline detergents (Delaval Alkali 1®, Ltd. Delaval, Gent, Belgium). In addition, the piglets of SG and CG received pelleted creep feed (Primary Choice®, Ltd. Boerries, Lindern, Germany) from the 7^th^ day of life. The consumed amount per litter was recorded daily. Moreover, the daily feed intake of the sows during lactation (13.2 MJ/kg), according to a feeding curve (Fig. 1), was documented per sensor. Litter sizes were standardised within 48 h *post partum*: SG sows retained as many piglets as they had functional teats, whereas CG sows retained one piglet less than they had functional teats. All piglets were cross-fostered only within their group. Surplus piglets were fostered by sows not taking part in the experiment in other farrowing rooms. During their first day of life, all piglets were weighed, tail-docked and received an iron injection. At the age of around four days, males were castrated.

### Assessed parameters

The ambient temperature in the farrowing room was recorded daily using one thermometer (Mini-/Maxi-Thermometer, Ltd. Egufer, Cologne, Germany) hanging freely at the height of the sows’ head. After analysing the measured stable temperatures, two seasons, “warm” and “cold”, were defined. The average temperature for the warm season (July to October 2011) was 24.7 °C, and for the cold season (October 2011 to April 2012) 21.3 °C. All piglets were weighed on days 1, 7, 14, and at the time of weaning. Additionally, piglet losses with causes (anomaly, crushed, born small sized, starved, other reasons), indication of medication (lameness, facial lesions, join lesions, inflammation e.g. panaritium, prophylaxis), and the occurrence of diarrhoea (severity graded into categories: 0 = no occurrence, 1 = slight, 2 = intermediate, 3 = severe) were documented daily. The BCS between 1.00 (skinny) and 5.00 (fat) at 0.25 intervals [10], and the BW of the sows were determined at housing-in and -out in the farrowing pens. On a weekly basis, BT was measured at three points [11] via ultrasound technique (Agroscan L®; Ltd. Hauptner und Herbolz, Solingen, Germany). After birth and at the time of weaning, the functionality and development of the sows’ udder as well as teat and udder skin injuries were evaluated according to an evaluation sheet (Table 3). On every day, the sows were examined clinically with special emphasis on the mammary glands to detect possible cases of mastitis. They were defined as affected by Postpartum-Dysgalactia-Syndrome and treated medicamentally when their rectal temperature was above 39.5 °C 24 h *post partum* and the mammary glands showed symptoms of inflammation. In addition, the appearance and the performance of the piglets were evaluated with regard to their behavior and body condition.

Furthermore, data of weaning-to-heat interval, and the pregnancy rate, defined as the percentage of litters born based on first inseminations [12], were recorded with regard to the consecutive parities. Additionally, samples of supplementary milk from the milk tank were taken for microbiological analyses between the 3^rd^ and 23^th^ day of lactation every five days, after circulating one day in the Supp-Le-Milk-System® (batch 11 to 15, *n* = 25). These milk samples were analysed bacteriologically using direct cultivation on Columbia sheep agar, incubation for 24 h at 37 °C, Gram staining and biochemical tests (API Staph®, bioMérieux, Nürtingen, Germany).

### Statistical analysis

Statistical analysis was performed using the software package SAS 9.2 (SAS Institute Inc. Cary, USA). The body weight of both sows and piglets, the number of piglets, difference in backfat thickness between housing-in and -out, and BCS of sows were examined using a generalised linear mixed model (Mixed-procedure) involving fixed effects of group (CG, SG), batch (1–15) and parity number (A (1,2), B (3,4), C (5–9)) as well as random effects (sow nested within parity). Lactation day (1–27) or week of lactation (week (1–4)) were used as covariates; lactation day was used to calculate piglet and sow weights and BCS, and week of lactation to calculate backfat thickness and the feed intake of the sows. To analyse piglet weight, the fixed effect of gender (male, female) and the random effect (piglet) was added. As the BT had different starting values in SG and CG despite random assignment to each group, differences in BT measured each week were used to examine differences between SG and CG. A mixed model was fitted using the restricted maximum likelihood method (REML). The results were expressed as least squares means (LSM). A Bonferroni correction was used for a multiple-significance-test correction. Piglet mortality, the medication rates of piglets, microbiological analyses and the occurrence of diarrhoea were analysed using the chi-square test (Freq-procedure). Furthermore, the significance of differences in the stable temperature was analysed with the Wilcoxon-Mann–Whitney test (Npar1way-procedure).

### Piglets’ performance and health

On average, 53.71 piglets per batch belonged to the supplemented group (SG) and to the control group (CG), respectively. For all batches, an average of 8.86 (±9.98) litres of supplementary milk was consumed per day in SG, corresponding to 1.06 (±1.20) kg milk powder per batch and day. Individual consumption of 0.02 (±0.02) kg milk powder per day and piglet was estimated on the basis of these data. Milk consumption increased continuously between the 2^nd^ day of lactation and the day of weaning. Moreover, the consumption of supplementary milk differed significantly between the different batches (*P* < 0.001), and the different seasons “warm” (1.49 kg/day and batch) and “cold” (0.85 kg/day and batch) (*P* < 0.001). SG piglets consumed significantly more creep feed than piglets of CG (7.34 (±15.18) g *v.* 5.87 (±12.44) g per day; *P* < 0.001). Creep feed consumption during the “warm” and the “cold” seasons did not differ (*P* > 0.05).

Equal numbers of piglets born alive in SG and CG were documented with an average birth weight of 1.3 kg per piglet (Table 1). Only gender showed a significant influence on the birth weight (*P* < 0.05) (Table 1). Under consideration of the performed litter size adjustments within 48 h *post partum,* SG and CG sows weaned 13.47 and 12.36 piglets (*P* < 0.05), respectively. All piglets showed equal weaning weights (Table 1) both in SG and in CG. The average daily weight gain did not differ significantly between SG and CG. However, a significant effect of the batch number on average daily weight gain *(P* < 0.05) was assessed. The total litter birth weight of SG did not differ significantly from the total litter birth weight of CG. The total weaning weight was significantly different between SG and CG. Additionally, the parity number showed a significant influence on the total weaning weight (Table 1). Overall, piglet losses occurred on average at the age of 2.4 days in SG, and at the age of 2.6 days in CG. Total losses over the whole suckling period did not differ significantly between SG (13.8 %) and CG (16.4 %) (p = 0.1). An analysis of the individual causes of mortality showed no significant differences between SG and CG with regard to crushing, low birth-weight, starvation or any other documented possible causes. A significant difference only appeared for anomalies *(P* < 0.05), which belong to innate causes of losses. The occurrence of diarrhoea did not differ significantly between SG and CG (*P* = 0.09) with regard to frequency of the assessed grades. Medicinal therapies of piglets did not differ significantly between SG and CG regarding the indications of lameness, infection prophylaxis, abrasion of the foreleg, or combinations of abrasion of the foreleg and facial lesions. However, piglets with signs of facial lesions alone were more often treated in CG than in SG (46 *v*. 32 treatments; *P* < 0.05).

**Table 1:** Effect of milk supplementation, sex and parity class on piglets’ traits

	Treatment		Sex		Parity class			RSD^a^	P-values		
	SG^b^	CG^c^	Male	Female	A (1/2)	B (3/4)	C (5–9)		Group	Sex	Parity class
Bodyweight at birth, kg	1.34	1.29	1.34^a^	1.30^b^	1.32	1.34	1.30	0.26	0.170	0.004	0.606
Bodyweight at weaning, kg	7.83	7.81	7.86	7.77	7.92	7.90	7.64	1.54	0.145	0.257	0.292
Bodyweight gain, g/d	245.2	245.5	246.0	244.7	249.9	249.0	237.0	2.8	0.942	0.627	0.115
Number of piglets born	16.78	16.82			17.09	16.73	16.58	3.32	0.947		0.869
Number of piglets weaned	13.47^a^	12.36^b^			13.06	13.09	12.65	1.03	<.0001		0.196
Litter weight at birth, kg	21.92	21.14			21.19	22.01	21.38	4.26	0.318		0.723
Litter weight at weaning, kg	105.13^a^	96.75^b^			103.68^a^	102.98^a^	96.17^b^	11.08	0.001		0.026

^a^RSD: residual standard deviation

^b^SG: supplemented group

^c^CG: control group

*a,b: values within group, sex or parity class with different superscripts differ significantly at *P* < 0.05 *t*-test with Bonferroni correction

### Discussion: piglets’ performance and health

Litter sizes had to be adjusted for reasons of animal welfare. It was argued, based on the farm’s experience that CG sows are not able to raise as many piglets as teats without negative effects on one and more piglets, and the sufficient milk intake for each piglet should be guaranteed. SG sows therefore had to raise one more piglet than CG sows. Under the provision of supplementary milk, the sows of SG managed this without negative effects, for instance piglet losses, and therefore, partly, but not exclusively, based on the litter adjustments, a higher number of piglets were weaned in SG. Consequently, the total weaning weight of litters from SG was significantly higher than in CG, mainly related to the study design. In contrast to the findings of Azain et al. [13], and Miller et al. [14], piglets’ individual weaning weights did not differ between SG and CG. This difference is mainly related to the different experimental designs of other studies with the same numbers of weaned piglets (11–13) in the supplemented and the control groups [13–16]. In the presented case, the equal weaning weights of piglets in SG, with one more piglet, and CG showed that feeding supplementary milk can balance the described negative effects of increasing litter sizes on average daily growth rates [17]. A closer look at the individual causes of losses demonstrated a significant difference between SG and CG only in losses resulting from anomalies. However, deaths due to anomalies mainly occur in the first two days of life, where 62 % of all piglet losses appear [18]. However, supplementary milk was not offered until the 2^nd^ day of lactation. Therefore, any effect can be ruled out. When starting with milk supplementation, piglets had to adapt to the system and the additional support of milk. Therefore, the intake of supplementary milk increased over the whole suckling period, as previously reported by King et al. [16]. Diarrhoea due to unknown causes tended to occur more often in SG piglets than in CG piglets. Dewey et al. [19] mentioned that the use of additional supplementary milk may increase diarrhoea due to a reduced intake of sows’ milk. Video observation may provide more information on suckling frequencies in the groups in future observations.. With regard to the indications of medication, facial lesions were treated significantly more often in CG, even though the number of piglets per sow was lower than in SG. This disagrees with the findings of Hansson and Lundeheim [20], assessing a significantly increased occurrence of facial lesions with larger litter sizes. The findings of the presented case might suggest a reduced struggle for the udder with the provision of supplementary milk. However, the verification of this statement requires further behavioural studies. As reported previously by Azain et al. [13], supplementary milk intake differed significantly between batches and between “warm” and “cold” seasons. As milk production decreases at high barn temperatures [21–23], piglets compensate for this deficit by drinking more supplementary milk [13]. SG piglets showed a higher feed intake of creep feed than CG piglets. This contradicts the observations of Baumann [21], who reported a decrease in creep feed intake of milk-supplemented piglets. A high creep feed intake of piglets during the suckling period has to be regarded positively, since a good nutritional condition prepares them for their post-weaning period [24]. Nevertheless, the relation between additional supplementary milk intake and an increased creep feed intake, and the effects on growth in the post-weaning period should be examined in further studies.

### Sows’ performance and health

The average feed consumption of both SG and CG sows was equal with 5.31 kg per day during lactation. The losses in body weight (BW) of sows from the time of housing-in to housing-out did not differ significantly between SG and CG. Only parity and batch number showed a significant influence on BW. The Body Condition Score (BCS) of sows did not differ significantly at the time of housing-in and at the time of housing-out, and the parity number of sows showed a significant effect on the BCS of sows at the time of housing-out (Table 2). With regard to the loss of backfat thickness (BT) during the suckling period, no significant differences occurred, as shown in Table 2. However, the week of measurement showed a significant influence (not shown). Sows exposed both treatments had a similar number of functional teats at farrowing (Table 3). At the time of weaning, SG sows showed significantly more functional teats than CG sows (Table 3). While the development of the udder was almost equal at the time of birth, the percentage of middle- to high-grade udder developments was higher in SG sows than in those of CG at the time of weaning. In contrast, the percentages of none- and low-grade udder developments were higher in CG than in SG. However, the occurrence of teat and udder skin injuries in SG sows was similar to that of CG sows at the time of birth and at housing-out as shown in Table 3. The duration of weaning-to-heat interval was four days for both SG and CG sows. The pregnancy rate did not differ significantly between SG and CG (SG: 88.7 % *v*. CG: 88.9 %; *P* > 0.05). With regard to the clinical occurrence of mastitis, no significant differences were detected. Five sows of SG (8.3 %) and three sows of CG (5.0 %, *P* > 0.05) became affected by the Postpartum-Dysgalactia-Syndrome followed by medication within three days *post partum*.

**Table 2:** Effects of group and parity class on sows’ condition (Least square means, RSD^a^, P-values)

	Treatment		Parity class				P-values	
	SG^b^	CG^c^	A (1/2)	B (3/4)	C (5–9)	RSD	Group	Parity class
Sow live weight, kg								
Housing-in	271.7	270.8	245.9^a^	269.0^b^	299.0^c^	14.5	0.728	<.0001
Housing-out	234.0	235.8	208.3^a^	232.0^b^	264.4^c^	18.9	0.492	<.0001
Body condition score^d^								
Housing-in	3.86	3.84	3.92	3.75	3.88	0.33	0.741	<.0001
Housing-out	2.92	2.89	2.75^a^	2.79^b^	3.17^c^	0.41	0.904	<.0001
Difference in backfat thickness Housing-in to -out, mm								
Anterior	3.76	4.71	4.99	4.11	3.60	2.40	0.061	0.196
Middle	2.91	3.26	3.69	2.96	2.62	1.17	0.353	0.136
Posterior	3.00	3.33	3.98^a^	3.00^a,b^	2.51^b^	1.84	0.351	0.037

^a^RSD: residual standard deviation

^b^SG: supplemented group

^c^CG: control group

^d^classes 1.00 (skinny) to 5.00 (fat), 0.25 intervals

*a,b: values within group or parity class with different superscripts differ significantly at *P* < 0.05 *t*-test with Bonferroni correction

**Table 3:** Evaluation of the udder at time of housing-in and housing-out

	Housing-in		Housing-out	
Treatment	SG^a^	CG^b^	SG	CG
Total number of sows	60	60	60	58
Functionality^c^				
Average number of functional teats per sow	14.0	14.2	13.5	12.8
Udder development^d^, %				
No development	0.7	0.4	7.7	11.1
Low-grade	5.2	4.2	9.4	10.4
Middle-grade	35.1	35.2	28.4	22.5
High-grade	58.9	60.3	54.6	56.1
Teat injuries^e^, %				
None	99.2	98.7	97.6	97.6
Slight	0.4	0.8	1.9	2.4
Intermediate	0.4	0.5	0.4	0
Severe	0.1	0.1	0.2	0
Udder skin injuries^f^, %				
None	92.2	91.7	89.6	89.6
Slight	7.8	8.3	9.5	9.4
Intermediate	-	-	0.9	0.9

^a^ *SG* supplemented group

^b^ *CG* control group

^c^number of data: 849 to 892 according to treatments

^d^number of data: 845 to 878 according to treatments; 4 classes: no to high-grade

^e^number of data: 849 to 892 according to treatments; 4 classes: no to severe

^f^number of data: 849 to 892 according to treatments; 3 classes: no to intermediate

### Discussion: sows’ performance and health

Sows were fed according to a feed curve and not *ad libitum*, the provision of supplementary milk for the piglets did not show any significant effect on the feed intake of sows. However, this result is in accordance with Azain et al. [13], who fed sows *ad libitum*. Similar to the results of King et al. [16] and Dunshea et al. [25], the decrease in BT did not differ between sows of SG and CG, although SG sows had to raise one more piglet in the current study. Auldist et al. [17] described a decrease in BT with increasing litter sizes with two more piglets in the larger litters, but without provision of supplementary milk.

With regard to the BW of sows, SG sows lost 2.63 kg more than CG sows, although no significant differences were found. This is in accordance with findings of Eissen et al. [26] and Kim and Easter [7], who described an increase in body weight loss with larger litter sizes. The udder evaluation at the time of weaning gave hints of better udder stimulation by the SG piglets compared to CG piglets. On the one hand, this might be related to the experimental design: SG sows had to raise one more piglet, which in turn led to an increase in milk production [2, 3, 17], and increased mammary gland growth [27]. On the other hand, SG piglets might be strengthened by the intake of supplementary milk and stimulate the milk production of the sow additionally [27].

### Hygiene of the system

The microbiological analysis of supplementary milk, after circulating one day through the piping system, mainly revealed *Enterobacteriaceae* in low numbers (in 96.0 % of all samples (*n* = 24)). *Enterobacteriaceae* suggest an environmental contamination, as described by Hadina et al. [28] for farm environments. *Staphylococcaceae* were isolated in 12.0 % (*n* = 3) of the samples*,* and *Streptococcaceae* in 4.0 % (*n* = 1)*.* There was no significant increase in bacteria recorded from the first to the last sampling (*P* > 0.05), indicating the effectiveness of the daily disinfection with peracetic acid, and the monthly disinfection with alkaline detergent.

## Conclusions

Despite rearing one more piglet, the growth of piglets remained stable, and the sows did not lose significantly more body substance in SG. In general, the provision of supplementary milk allows the raising of more piglets on and in direct contact with the sow. However, the maximal number of weaned piglets per sow clearly depends on the individual farm management. Therefore, as shown by the presented case, supplementary milk provision in the farrowing pen represents a useful tool when managed properly and adapted to the conditions and demands of the single farms.

## Abbreviations

Cm: Centimetre
g: Gramme
h: Hours
kg: Kilogramme
m^2^: Square metre
MJ: Megajoule
*v*.: *Versus*
%: Percent
